# Molecular characterization of hemagglutinin-neuraminidase fragment gene of Newcastle disease virus isolated from periodically-vaccinated farms

**DOI:** 10.14202/vetworld.2018.657-666

**Published:** 2018-05-20

**Authors:** Lucia S. Triosanti, Michael Haryadi Wibowo, Rini Widayanti

**Affiliations:** 1 Department of Microbiology, Faculty of Veterinary Medicine, Gadjah Mada University, Yogyakarta, Indonesia; 2 Department of Biochemistry, Faculty of Veterinary Medicine, Gadjah Mada University, Yogyakarta, Indonesia

**Keywords:** hemagglutinin-neuraminidase, Newcastle disease, protein, reverse transcriptase polymerase chain reaction, sequencing, vaccination, virus

## Abstract

**Background and Aim:**

Newcastle disease (ND) caused by avian paramyxovirus serotype-1 (APMV-1) is long known as an acute contagious and infectious disease of various bird species. Prior studies have acknowledged that the virus could cause up to 100% morbidity and mortality as well as reducing eggs production. In theory, hemagglutinin-neuraminidase (HN) in ND virus (NDV) is one of the surface glycoproteins that functions during the attachment, assembly, and maturation of the virus. On the fields, Indonesia has been recognized as an endemic country for ND where continuous outbreaks of ND in commercial chicken farms have been reported despite the implementation of periodical vaccination programs. Thus, this study aims at characterizing NDV isolated from periodically vaccinated commercial farms, comparing its genetic correlation based on their HN gene fragment with registered NDV originated from Indonesia as well as with existing vaccine strains.

**Materials and Methods:**

The HN gene fragment of NDV isolated from well-vaccinated farms was amplified using primer pairs of forward 5’ GTGAGTGCAACCCCTTTAGGTTGT 3’ and reverse 3’ TAGACCCCAGTGATGCATGAGTTG 3’ with a 694 bp product length. The nucleotide sequences of nine samples, which were gathered from Kulon Progo, Gunung Kidul (2), Boyolali (2), Magelang, Muntilan (2), Palembang, and Medan, were later compared with the sequences of HN gene of NDV available in NCBI Genbank database. The amino acid sequence analysis and multiple sequence alignment were conducted using the Mega7 program.

**Result:**

The data analysis on amino acid sequences showed that the structure of amino acid residue at positions 345-353 for all isolates appears to be PDEQDYQIR. The structure is the same as for archived samples from Indonesia and either LaSota or B1 vaccine strains. The amino acid distance between observed isolates and LaSota vaccine strain is 8.2-8.8% with a homology value at 91.2-91.7%.

**Conclusion:**

Looking at amino acid sequence analysis, LaSota vaccines can considerably be stated as being protective against ND disease outbreak. However, the distant homology value from a perfect condition for the protection might have acted as the root cause of vaccination failures.

## Introduction

Newcastle disease (ND; known as *tetelo* disease in Indonesia) has been reported as critically affecting the economics of poultry industry [1]. In fact, the virus has caused numerous economic losses in the industry including morbidity and mortality rates that may reach up to 100% along with decreased egg productions [1,2]. In the world, the ND virus (NDV) worldwide spread and high transmission have even made the disease to get included in the LISTED (reportable disease) by World Organization for Animal Health [3]. At the national level, ND has been reported in Indonesia as an endemic disease because its cases have been reported throughout the year in various regions [4].

In Indonesia, an intensive vaccination in conjunction with good management of chicken farms has been considered as a comprehensive strategy to prevent outbreaks of ND [5,6]. In particular, various vaccination programs and commercial type vaccines (either living or killed vaccines) have been applied in Indonesia. In the field practice, vaccinations are conducted either independently for a single disease (ND) or in combination to prevent a set of different poultry diseases [7].

Despite a continuous implementation of the vaccination programs, ND still stands as a major problem in the poultry industry, in which numerous cases of ND are continuously reported in periodically vaccinated chicken farms. Despite the critical importance of hemagglutinin-neuraminidase (HN) as a surface glycoprotein during the attachment, assembly and maturation of NDV, however, there is no report on the characterization of HN in NDV gene isolated from Indonesia. Therefore, this study first aims at characterizing outbreak-causing NDV based on the HN gene fragment. To investigate the cause of continuous occurring of ND despite a consistent implementation of vaccination programs, sample viruses need to be isolated from well-vaccinated commercial poultry farms. The results will be the first scientific report on the characteristics of HN gene isolate virus from Indonesia.

Then, this study was proposed to observe any genetic correlation between observed samples isolated in this study, existing isolated samples from Indonesia that have been previously recorded at GenBank database and the strain of LaSota vaccine.

## Literature Review

The NDV was first recorded to cause an outbreak in 1926 in Java Island, Indonesia [8], and in 1927 near Newcastle-upon-Tyne, England [9]. Years later, ND subsequently spread and became endemic in many countries [2,10]. In Asia, Africa and Central as well as South America, the epizootics of ND in poultry continued to occur, while sporadic epizootics occurred in Europe [11,12]. During 2009-2010, there was a report of ND outbreaks that occurred at various commercial chicken farms in Indonesia, resulting in an around 80% mortality rate [13]. The morbidity and mortality rates in different flocks were in fact varied, following the variation of respective NDV strains. According to Darminto and Ronohardjo [14], losses that might considerably be related to the continuous occurrences of ND disease in Indonesia have been estimated to reach beyond US$10 million annually.

In the literature, ND disease has been noted to be caused by avian paramyxovirus serotype-1 (APMV-1), which belongs to family *Paramyxoviridae*, genus *Avulavirus*, Avian paramyxovirus serogroup [15]. In details, the virus has a non-segmented, negative sense, and single-stranded RNA genome [16,17]. Regarding its size, the NDV genome has an approximately 15.2 kb in length [18,19]. Regarding its content, the virus genome encodes six structural proteins including nucleoprotein (NP), phosphoprotein (P), matrix (M), fusion protein (F), HN and dependent RNA polymerase (L). Besides, there are two non-structural proteins (V and W proteins) produced during the transcription of P gene. HN and F proteins are located on the virus membrane surface, while N, P and L proteins form the genetic material complex of the virus. Practically, NDV proteins deliver the virulence within a host’s body [20]. First, an NDV infection is initiated by a receptor recognition and the virion binding to sialoglycoconjugates on the host cell surface. The binding process is followed by a fusion of the viral lipid envelope with cell host membrane [21]. Then, the interaction of two viral surface glycoproteins, i.e. HN and F, accomplishes the NDV infection process [22].

In particular, HN protein has been recognized as having a critical contribution during NDV pathogenesis [23,24]. The protein delivers their multifunction in the attachment process between virus and host. In the release of virus from an infected cell, it produces neuraminidase (NA) activity [21]. During the attachment process of NDV to a host cell, HN protein acts as an agent to recognize a sialic acid receptor on the surface of targeted host cells. Besides, the protein triggers a fusion process of viruses in target cell membranes in conjunction with F protein. Theoretically, F protein requires cooperation with HN protein to perform a membrane fusion during the fusion process so that the virus may penetrate a host cell’s surface [25,26].

Furthermore, HN protein has discovered to vary regarding its length. In details, nine different variants have been reported, i.e. 570 amino acids (570aa), 571aa, 572aa, 577aa, 578aa, 586aa, 582aa, and 616aa [19,27]. The length of HN protein, however, does not affect the virulence of NDV [28]. Within itself, HN protein has five antigenic sites, i.e. amino acid residues 193-201, 345-353, 494, 513-521, and 569 [29]. In theory, HN protein has been known to have a critical role in inducing immunity against different viral infections. However, the protein is susceptible to the immune defense system of an infected individual host. The vulnerability during an encounter then makes the protein to simultaneously response by straightforwardly producing antigenic variations [29,30]. In particular, the amino acid residue at position 347 in HN protein is critical for virus recognition reaction by monoclonal antibodies against HN [31]. Their sequence analysis has shown that a mutation in amino acid residue 347 from E to K would change the character of the HN protein and make its escape from epitope recognition by monoclonal antibodies. Cho *et al*. [32] have reported that the occurrence of E347K mutation in NDV HN protein may reach up to 87.5%. The high prevalence of the mutation could serve as a marker for antigenic variations in NDV.

In general, hygienic treatments and vaccination are two commonly-taken approaches to control NDV spread in commercial farms. The former, i.e. hygienic treatments, includes several techniques such as cleaning, disinfecting, limiting accesses to wild birds, and strict guidance for personal/individual hygienic behavior of farm staffs [33]. The latter, i.e. vaccination, is known to provide an excellent means to decrease chances of clinical disease caused by a virulent NDV [34]. According to Fentie *et al*. [35], Shabbir *et al*. [36] and Yune and Abdela [37], a progressive vaccination program should be implemented in combination with biosecurity actions to control the disease. However, vaccination-related policies may vary in different countries [16]. In Indonesia, various vaccination programs have been implemented, which consist of a combination of activated and inactivated ND vaccines. A prior study by Wibowo and Amanu [6] has proven that chickens treated with the combination of both types of vaccine are more effectively protected compared to those receiving activated vaccines only. In general, LaSota and B1, which were first developed based on lentogenic NDV strains, are the most commonly used vaccine strains worldwide [38]. In Indonesia, data from the country’s Center for Quality Testing and Certification of Veterinary Drugs (BPBPMSOH; *Balai Besar Pengujian Mutu dan Sertifikasi Obat Hewan*) have shown that vaccines registered in Indonesia include LaSota, B1, RIVS 2, Clone 30, ITA, Mukteswar, Kimber, Ulster, and Genotype 7 (G7) [39]. In general, each of these vaccines is primarily designed to have a full capability in delivering a protection against ND infections if the vaccine is in a viable condition, if it is administered through a proper set of procedures to healthy birds by following applicable guidance, and if there is enough time frame to form an immune response by vaccinated birds to counter an exposure to the virus [40].

## Materials and Methods

### Observed samples

This study investigates field samples taken from laboratory collections, which were previously gathered from various areas and sent to Microbiology Laboratory at the Faculty of Veterinary Medicine, Gadjah Mada University (UGM), Yogyakarta, Indonesia. Among the laboratory collections, a total of nine samples were selected including those gathered from ND outbreak-occurring areas of commercial chicken farms (Table-1).

**Table-1:** Observed samples in this study.

No	Sample code	Origin	Year	Poultry
1	ND-Lay/KP-145/2013	Kulonprogo	2013	Layer
2	ND-Lay/GK-SR1/2013	Gunungkidul	2013	Layer
3	ND-Lay/GK-SR2/2013	Gunungkidul	2013	Layer
4	ND-Lay/BYL-1/2014	Boyolali	2014	Layer
5	ND-Lay/BYL-3/2014	Boyolali	2014	Layer
6	ND-Lay/MGL-Pullet-80/2013	Magelang	2014	Layer
7	ND-Bro/MNL-Lingga-2L/2014	Muntilan	2014	Broiler
8	ND-Lay/Medan-Pullet-KB/2014	Medan	2014	Layer
9	ND-Lay/PLB-147/2014	Palembang	2014	Layer
10	ND-Bro/MNL-Lingga-IP/2014	Muntilan	2014	Broiler

ND: Newcastle disease

### Isolation of NDV

Viruses were isolated by applying the OIE 2.3.14 protocol [41]. First, eggs produced by specific-pathogen-free chickens were gathered. When their embryos have been aged 9-11 days, the embryonated eggs were candled to check the viability of embryos. Next, viable eggs were first pre-conditioned in an incubator for 2 days at 37°C to put them in good condition before being inoculated. In parallel, sample organs (pulmonary and brain) of ND-infected chickens were homogenized using tissue raptor to have a homogeneous form. The supernatant of the homogenized sample organs was taken as inoculating NDV. After that, the pre-conditioned viable eggs were inoculated by injecting the prepared inoculating NDV as much as 0.1 ml to chorioallantoic fluid (CAF) of the eggs using a 1 ml syringe. Next, the inoculated eggs were incubated at 37°C. Later, a candling process was repeatedly performed over these incubated eggs. The observation on their embryos was conducted once every 24 h. If an observation revealed the embryonic death of an egg, the respective egg was moved and maintained in a refrigerator. After all incubated eggs have shown embryonic deaths, the CAF of each egg was harvested separately.

### Virus identification

#### Hemagglutination (HA) test

A total of 0.025 ml phosphate buffer saline (PBS) was placed on a plastic-made microtiter plate (U-shaped well). Next, a 0.025 ml of viral suspension was added to the first well. The virus suspension was diluted into the last well by a multichannel micropipette. In each well, a 0.025 ml PBS solution was added. Besides, a total of 0.025 ml of 0.5% random chicken red blood cells was added to each well. The red blood cells-added plates were placed at room temperature and observed after 30 min. Alternatively, the plates were placed at 20°C temperature and observed after 60 min.

#### Hemagglutination inhibition (HI) test

For HI test, a 0.025 ml PBS solution was added to a microtiter plate. Wells with a U-shaped base, except for the first well, acted as a 4-HAU control line. Next, a 0.025 ml ND-specific antiserum was added to the first well, which was then diluted two-fold to the next until last wells for the titration using a multichannel micropipette. After that, a 0.0.25 ml 4HA virus/antigen was added into each well. The plates were then placed at room temperature for 30 min. Alternatively, they might be placed at 4°C temperature for 60 min. The next step was the addition of 0.025 ml of 0.5% random chicken red blood cells into each well. The plates were again incubated for 40 min at room temperature. Alternatively, they might be incubated for 60 min at 4°C temperature. Furthermore, the incubation could be stated as finished after red-blood cell control had been agglutinated.

#### Reverse transcriptase polymerase chain reaction (RT-PCR)

RNA extraction was conducted on a 200 µl of CAF using Purelink Viral RNA/DNA mini-kit by following suggested protocols from the kit’s manufacturer. After that, the RNA extraction results were taken into the RT-PCR process using forward and reverse primers. The process was conducted to amplify the HN sequence of NDV (Table-2).

**Table-2:** Designed primers (forward and reverse) at nucleotide positions 659-1353 of HN gene.

Target	Primers	Nucleotide	Length	Positions
HN	Forward	5’GTGAGTGCAACCCCTTTAGGTTGT3’	694 bp	659
	Reverse	5’TAGACCCCAGTGATGCATGAGTTG3’		1353

HN: Hemagglutinin-neuraminidase

In details, the primer was designed to amplify nucleotide residues at positions 659-1353 (amino acid 256-449) of HN protein using AmplifiX software. Amplification areas were selected according to the immunodominant area of HN protein of NDVs. In particular, amino acid residues at positions 345-353 have been recognized as having a critical role during the antigenic formation of NDV. Primary specificity of the primer was tested according to Blast search from the National Center for Biotechnology Information (NCBI). Next, aforementioned samples and the primer were replicated and transformed into cDNA at 50°C for 30 min. After that, the produced cDNA was pre-denaturized at 95°C for 2 min. The pre-denaturation step was performed for 1 cycle only. Next, the pre-denaturation results were denaturized at 95°C for 15 s. The denaturation results were annealed at 59°C for 30 min and were extended at 68°C for 60 s. These three processes (denaturation, annealing and extension) were repeated for 35 cycles. Next, results produced after these 35 cycles were taken to post-extension steps at 72°C for 5 min. The post-extension steps were performed for 1 cycle only, and the machine was set to automatically hold at 4°C as a safety precaution for preventing undesired damages on the amplified fragment gene. After all of these RT-PCR cycles have been completed, a gel electrophoresis process of the RT-PCR product was performed using a 1.5% agarose gel with Florosafe Stain DNA stain in a 1× buffer TBE solution. Next, the RT-PCR amplification product in the gel was visualized using UV transilluminator. Then, the RT-PCR product showing positive results was taken to FIRST BASE Laboratory in Singapore for nucleic acid sequencing using a capillary sequencer.

### Data analysis

From the nucleic acid sequencing, the sequence result/data were received as an electropherogram in the form of ABI file. The data were converted into a txt file containing the nucleic acid sequence. Next, the converted sequence data were assembled. It was followed by searches on NCBI databases to find similar sequence(s). Next, an analysis of the sequencing data was conducted using Molecular Evolution Genetics Analysis (MEGA) software version 7 [42,43]. In details, the sequence searches were followed by a multiple sequence alignment using MEGA7’s ClustalW tool. Genetic distance and homology value of amino acids and nucleotide were analyzed by applying “p-distance” and “no. off difference” options in the program. Next, the data were analyzed using ClustalW’s “Neighbor-Joining” option to obtain a phylogenetic tree. Then, the replication percentage of branches-forming phylogenetic tree was tested using a bootstrap test on 1000 replications.

## Results

### Isolation and identification of NDV

**Figure-1:**
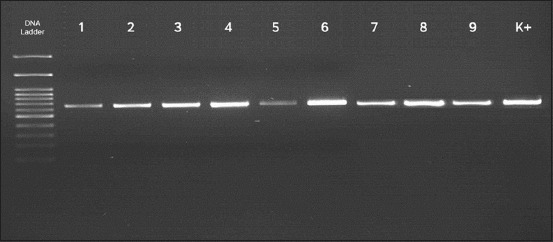
Electrophoresis of HN gene fragment amplification. Samples 1-9 show positive results, looking at considerably parallel band positions to the control (K+). In this study, LaSota vaccine strain is posited as the positive control.

NDV isolation was performed by injecting the supernatant of homogenized pulmonary and brain organs from suspected chicken samples of NDV into the CAF of specific-pathogen-free, embryonated and viable eggs. The NDV isolates would cause the death of chicken embryos with an inoculation time of approximately 28 h. The CAF from the inoculated chicken eggs was then used for viral identification tests. Apparently, HA tests showed that all samples appeared to have positive results. Next, the samples showing positive were again tested for HI using a specific anti-ND serum. Apparently, the HI tests showed positive results for NDV also. The nine samples under observation, therefore, showed positive in both HA and HI tests. Table-3 provided the results of isolation and virus identification conducted in Microbiology Laboratory at the Faculty of Veterinary Medicine (FKH), Gadjah Mada University (UGM). Furthermore, a molecular test was conducted by implementing RT-PCR method on samples that showed positive results during both HA and HI tests. The electrophoresis results showed the presence of samples’ nucleic acid bands (1-9) as being parallel to the positive control (LaSota vaccine, K+) at 694 bp (Figure-1).

**Table-3:** Vaccination records, clinical signs, the results of HA, HI, and RT-PCR test on observed samples.

No	Samples code	Vaccination	Clinical signs	Test results		

				HA	HI	RT-PCR
1	ND-Lay/KP-145/2013	4 (ND IB live); 18 (ND live); 35 (ND live)	Production drop, torticollis, mortality 5%	+	+	+
2	ND-Lay/GK-SR-1/2013	16 weeks, revaccination: 1.5 month	Production drop	+	+	+
3	ND-Lay/GK-SR2/2013	16 weeks, revaccination: 1.5 month	Production drop	+	+	+
4	ND-Lay/BYL-1/2014	1 (ND IB live); 5 (ND IB killed); 27 (ND live); 49 (ND IB live); 70 (ND IB live)	Torticollis, high mortality	+	+	+
5	ND-Lay/BYL-3/2014	1 (ND IB live); 5 (ND IB killed); 27 (ND live); 49 (ND IB live); 70 (ND IB live)	Torticollis, high mortality	+	+	+
6	ND-Lay/MGL-Pullet-KB/2014	4 (ND IB); 21 (ND live); 56 (ND IB)	Production drop, High mortality	+	+	+
7	ND-Bro/MNL-Lingga-2L/2014	4 (ND IB live); 21 (ND)	High mortality	+	+	+
8	ND-Lay/PLB-147/2014	5 (ND live); 16 (ND live); 28 (ND live); 35 (ND killed)	High mortality	+	+	+
9	ND-Lay/Medan-Pullet-KB/2014	1 (ND IB); 4 (ND IB killed); 20 (ND live)	High mortality	+	+	+

HA: Hemagglutination, HI: Hemagglutination inhibition, RT-PCR: Reverse transcriptase-polymerase chain reaction, ND: Newcastle disease

### Sequence analysis on NDV HN fragment gene (amino acids 256-449)

For nine samples under observation, the amino acid sequence at positions 345-353, which were known as the immunodominant area of HN gene of NDV, was discovered to be ^345^Proline- ^346^Aspartate- ^347^Glutamate- ^348^Glutamine- ^349^Aspartate- ^350^Tyrosine-^351^Glutamine- ^352^Isoleucine- ^353^Arginine (PDEQDYQIR). Table-4 provides the composition of amino acid residues at positions 345-353 of the nine samples under investigation, existing samples of NDV isolates from Indonesia previously recorded in the GenBank database, and existing vaccine strains used in Indonesia. It appeared that all of these current, existing and vaccine samples have the same amino acid sequence of PDEQDYQIR. The complete comparison of amino acid changes in individual virus isolates is provided in Table-5.

**Table-4:** Immunodominant area from observed samples, previously-recorded isolates from Indonesia in GenBank, and the LaSota strain.

Virus	Genbank accession number	Origin	Residue (345-353)
**Samples**			
ND-Lay/KP-145/2013	-	Kulonprogo	PDEQDYQIR
ND-Lay/GK-SR-1/2013	-	Gunung Kidul	PDEQDYQIR
ND-Lay/GK-SR2/2013	-	Gunung Kidul	PDEQDYQIR
ND-Lay/BYL-1/2014	-	Boyolali	PDEQDYQIR
ND-Lay/BYL-3/2014	-	Boyolali	PDEQDYQIR
ND-Lay/MGL-Pullet-KB/2014	-	Magelang	PDEQDYQIR
ND-Bro/MNL-Lingga-2L/2014	-	Muntilan	PDEQDYQIR
ND-Lay/PLB-147/2014	-	Palembang	PDEQDYQIR
ND-Lay/Medan-Pullet-KB/2014	-	Medan	PDEQDYQIR

**Previous Isolates from Indonesia**			

NDV/Cockatoo/Indonesia/14698/90	AY288998	Indonesia	PDEQDYQIR
NDV/Chicken/Bali/020/10	HQ697261	Bali	PDEQDYQIR
NDV/Chicken/Banjarmasin/010/10	HQ697254	Banjarmasin	PDEQDYQIR
NDV/Chicken/Gianyar/013/10	HQ697257	Bali	PDEQDYQIR
NDV/Chicken/Kudus/017/10	HQ697259	Kudus	PDEQDYQIR
NDV/Chicken/Kudus/018/10	HQ697260	Kudus	PDEQDYQIR
NDV/Chicken/Makassar/003/09	HQ697256	Makassar	PDEQDYQIR
NDV/Chicken/Sragen/014/10	HQ697258	Sragen	PDEQDYQIR
NDV/Chicken/Sukorejo/019/10	HQ697255	Sukorejo	PDEQDYQIR
NDV/Chicken/Jakarta/1997	JX393313	Jakarta	PDEQDYQIR

**Vaccine**			

LaSota	FJ004152		PDEQDYQIR

ND: Newcastle disease, NDV: Newcastle disease virus

**Table-5:** Differences between amino acid from isolate samples compared to vaccine strain LaSota.

No	Isolates	Amino acid positions															

		265	267	268	271	284	290	292	295	310	312	317	331	371	389	397	433
**Vaccine strain**																	

1	FJ004152_ND_LaSota	N	A	V	R	H	V	T	G	I	S	S	V	I	V	V	V

**Isolate samples**																	

2	ND-Bro/MNL-Lingga-2L/2014	K	I	A	S	R	T	A	K	V	E	P	A	V	M	I	I
3	ND-Lay/BYL-1/2014	K	V	A	S	H	T	A	E	V	E	P	A	V	M	I	I
4	ND-Lay/BYL-3/2014	K	I	A	S	R	T	A	K	V	E	P	A	V	M	I	I
5	ND-Lay/GK-SR1/2013	K	V	A	S	H	T	A	E	V	E	P	A	V	M	I	I
6	ND-Lay/GK-SR2/2013	K	V	A	S	H	T	A	E	V	E	P	A	V	M	I	I
7	ND-Lay/KP-145/2013	K	V	A	S	H	T	A	E	V	E	P	A	V	M	I	I
8	ND-Lay/Medan-Pullet-KB/2014	K	V	A	S	H	T	A	E	V	E	P	A	V	M	I	I
9	ND-Lay/MGL-Pullet-80/2013	K	V	A	S	H	T	A	E	V	E	P	A	V	M	I	I
10	ND-Lay/PLB-147/2014	K	I	A	S	R	T	A	K	V	E	P	A	V	M	I	I

ND: Newcastle disease

### Phylogenetic analysis of NDV

Phylogenetic analysis was performed on the fragment of NDV HN gene sequence. Looking at phylogenetic tree produced by an analysis on amino acids at positions 256-449, all isolates observed in this study, which were gathered from Kulon Progo, Gunung Kidul (2), Boyolali (2), Magelang, Muntilan (2), Palembang and Medan, were discovered to have a close relation with existing NDVs that had infected poultry farms in some regions in Indonesia, i.e. Banjarmasin (Accession: HQ697254), Gianyar (Accession: HQ697257), Kudus (Accession: HQ697259, HQ697260) and Sragen (Accession: HQ697258).

### Genetic distance and homology between HN gene fragments

Amino acids analyses on the nine virus isolates under observation in this study showed that their genetic distance was 0-1.5% with homology values at 98.4-100%. On the other hand, the genetic distance of amino acids between the nine virus isolates compared to previously recorded isolates from Indonesia (GenBank database) was 0-10.8% with homology values at 89.1-100%. Then, comparing NDV isolates under investigation with existing LaSota vaccine strain applied in Indonesia showed their amino acid genetic distance in between 8.2% and 8.8% with homology values at 91.2-91.7%. On the other hand, a nucleotide analysis on the nine virus isolates provided 0-1.2% genetic distance with homology values at 98.8-100%. Besides, the nucleotide distance between the observed virus isolates compared to previously recorded isolates from Indonesia (GenBank) was 0-7.7% with homology values at 92.3-99.8%. Then, comparing isolated NDV under investigation with existing LaSota vaccine strain discovered their nucleotide genetic distance to be in between 16.7 and 17% with 83.03-83.33% homology values.

## Discussion

Virus isolation remains the definitive diagnostic evaluation for NDV [41]. In this study, the nine virus isolates under observation were classified into the velogenic NDV group [1] because the embryonic deaths of inoculated egg samples were discovered to occur in less than 60 h. On the other hand, the HA and HI tests were performed as conventional evaluations for NDV identification. In fact, both HA and HI tests showed positive results for all samples. In particular, the positive results (+) in HA test were observed by discovering the agglutination of poultry red-blood cells at the bottom of observed wells plate. The agglutination could occur because the red blood cells were bound into virus particles. The process was assisted by hemagglutinin protein on the surface of the NDV. The protein has been recognized as having an ability to agglutinate red-blood cells [44]. In parallel, positive results discovered by HI tests were discovered by observing the formation of red blood cell precipitates at the bottom of the plate. These precipitates were formed due to the binding of viral suspension into specific serum of NDV. The binding process has been known to cause the NDV to not being able to agglutinate the red blood cells.

Furthermore, the RT-PCR procedures were conducted to identify virus from observed samples based on a molecular technique. Amplification was performed on HN gene fragments using primers designed in AmplifiX software. The presence of DNA bands, which appeared to be nearly parallel to the positive control (LaSota vaccine) at around position 694 bp, showed that the nine test samples were molecularly proven to be NDV. Besides, the pattern of amino acid residues at positions 345-353 of either the nine samples being tested, existing NDV isolates from Indonesia in the GenBank database, or the LaSota vaccine strains have the same amino acid sequence of PDEQDYQIR. The amplification of the amplified area was implemented according to prior researches conducted by Iorio *et al*. [29], Hu *et al*. [31], and Gu *et al*. [45]. These studies have suggested that the amino acid residue at positions 345-353 in the NDV HN protein was a critical immunodominant area during an antibody recognition process. There was, in fact, no significant difference regarding amino acid sequence between viral isolates and existing vaccine strains applied in Indonesia. Miller and Koch [46] have reported that a matched vaccine strain to an outbreak has the highest potential to provide superior protection against virus transmissions. However, multiple factors might decrease the effectiveness of vaccination. According to Nakamura *et al*. [47], a vaccine failure might occur due to numerous factors occurred within a host (immunosuppressive condition), in a vaccine itself (improper vaccination) and the occurrence of pathogenic virus. To complement a vaccination program, controlling NDV should be supported by a strict biosecurity system to holistically prevent the virus from ever in contact with a poultry farm [46]. Furthermore, previous studies by Iorio *et al*. [29], Hu *et al*. [31] and Gu *et al*. [45] on the immunodominant nature of HN protein have shown that existing vaccines used in Indonesia should be protective against any NDV outbreak. The absence of mutations at the immunodominant areas of NDV has caused antibodies of individual hosts produced by the vaccine to recognize the immunodominant areas [31]. Theoretically, the recognition of a host’s antibodies to the immunodominant region would lead to HA processes. The process of viral binding with the host’s cell receptors could hence be avoided. However, the protective characteristics of LaSota vaccine, in addition to evaluation on the amino acid sequence within an antibody recognition site, should also be evaluated from the genetic and homologous distances between a circulating virus and the vaccine.

Moreover, both HN and F proteins have been noted as being the main targets for the formation of different ND vaccines [46]. Besides, these two proteins have been posited as the primary target of a host’s immune response to ND. In particular, HN proteins would induce an immune response as the host’s body activated its defense against a viral infection. Therefore, the protein could easily produce an antigenic variation in NDV when confronted with the host’s immune system [38,48]. Cho *et al*. [32] have reported that the prevalence of amino acid residues of E347K in NDV occurred to be at 87.5%. Most of which were found in isolates circulating in Korea and China mainland and included in the genotype II and VII of NDVs. According to Zhu *et al*. [49], LaSota has been discovered to not being able to provide complete protection against an E347K infection. Hence, LaSota-vaccinated birds were still able to get infected and to shed the virulent challenge (infecting) virus. In this study, however, no isolate experienced any mutation on E347K amino acid residues.

**Figure-2:**
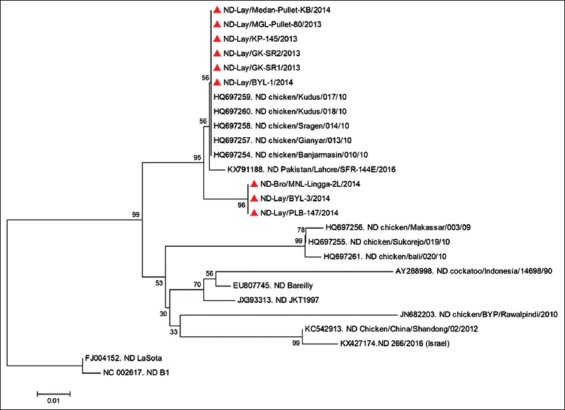
Phylogenetic tree of the HN fragment of the Newcastle disease virus based on amino acid structure at positions 256-449.

Looking at the phylogenetic tree produced by an analysis on amino acids (Figure-2), all nine isolates observed in this study, which were gathered from Kulon Progo, Gunung Kidul (2), Boyolali (2), Magelang, Muntilan (2), Palembang and Medan regions, have a close relation to previously-reported NDV isolates from Indonesia, which were recorded in the GenBank database. The existing isolates included that of Banjarmasin (Accession: HQ697254), Gianyar (Accession: HQ697257), Kudus (Accession: HQ697259, HQ697260) and Sragen (Accession: HQ697258). The last reported infection of these existing virus isolates occurred in 2010 [13]. Besides, an analysis on F gene conducted by Xiao *et al*. [13] has indicated that NDV isolated from Banjarmasin, Gianyar, Kudus and Sragen were classified into genotype VII. In addition, the nine observed virus samples also appeared to be closely related to isolates originated from Pakistan (Accession: KX427174), which was reported by Reihmani *et al*. [50] as having caused outbreaks in the country. Based on an analysis of its F genes, the Pakistan isolate was classified into genotype VII [50,51]. On the other hand, LaSota was classified as a strain of genotype II. Liu *et al*. [52] and Jeon *et al*. [53] have reported the LaSota (genotype II) vaccines (both live and killed vaccines) to deliver effective protection against viruses originated from different genotypes. However, Dimitry *et al*. [12] and Liu *et al*. [52] have reported vaccines that were phylogenetically close to outbreak-causing NDVs to provide better protection against ND. In particular, protection as such was observed to occur in terms of reducing the spread of NDV by infected individuals. Although conventional vaccines (LaSota) have been noted to work well in protecting poultry, it could not perfectly prevent infections and virus shedding from occurring [54,55].

Then, genetic distances discovered in this study would arguably affect the efficacy of any ND vaccination. In other words, it might alter a general consensus in which vaccines that were genetically matched with outbreaks virus would result in better protection against ND disease. In details, the lower the genetic distance between a virus on the field and a vaccine strain, protection produced against ND should be better. In this study, an investigation on the homology values between observed virus and LaSota vaccine strain revealed a considerably distant result compared to a perfect condition in which the vaccine had been tested as a perfect and consistent solution [12,56]. In short, a distance to the perfect condition has indicated a potential to allow an ND outbreak to occur even in periodically vaccinated commercial chicken farms. The argument has in fact supported by Yang *et al*. [57], Ewies *et al*. [58], Garcia *et al*. [59] and Mohamed *et al*. [60] who have noted that any genetic variation between a vaccine strain and a circulating NDV might cause the inability of currently used vaccine to deliver a protection against ND.

## Conclusion

Looking at the results of performed HN protein fragment analysis in this study, NDVs isolated from regularly-vaccinated commercial chicken farms appear to have an amino acid structure of PDEQDYQIR at residue positions 345-353. Besides, an analysis on the phylogenetic tree of the nine observed isolates has revealed a close relation to several previously recorded isolates from Indonesia (Banjarmasin, Gianyar, Kudus and Sragen) and Pakistan. Looking at various well-established vaccination programs implemented in chicken farms in Indonesia and at the amino acids structure of circulating NDVs, this study indicates that existing LaSota vaccines should be able to deliver effective protection against ND outbreaks in well-vaccinated commercial chicken farms. However, the discovered genetic distance between currently circulating virus and the LaSota vaccine strain might have acted as the probable root cause of ND outbreaks occurring in commercial chicken farms despite the regular implementation of various vaccination programs. The argument being proposed has in fact been supported by prior researchers. Those studies have underlined as small as possible differences between current virus strain condition by which a virus is circulating with the perfect virus strain condition to which a vaccine was first developed to deliver perfect protection as a critical cause of vaccination failures.

Then, prior study by Sarcheshmei *et al*. [61] has reported that any vaccination failure against a circulating NDV might also occur due to other factors such as the presence of immunosuppressive etiologic agents causing a lack of or inadequate responses to the vaccine; vaccination failures including an improper handling of vaccine administration; poor management practices in poultry flocks, *etc*. Therefore, further researches may put these factors as the focuses of studies to complement the current study that has suggested the lack of potency of a vaccine product as the probable root cause of vaccination failures.

## Authors’ Contributions

LST worked out a major part of the technical details, performed laboratory and computational preparations, tests as well as analyses, and took the lead in writing the manuscript in consultation with MHW and RW. MHW devised the project, conceived the research design, handled collaborative works with external partners, verified computational framework, and verified the results of laboratory and computational tests as well as data analyses. RW designed primers and computational framework, verified laboratory and data analysis methods and analyzed test results conducted by external partners. All authors read and approved the final manuscript.
